# Advances in technology and applications of nanoimmunotherapy for cancer

**DOI:** 10.1186/s40364-021-00321-9

**Published:** 2021-08-21

**Authors:** Lei Dou, Xiangdan Meng, Huiyuan Yang, Haifeng Dong

**Affiliations:** 1 Department of Gerontology, Tongji Hospital, Tongji Medical college, Huazhong University of Science and Technology, Wuhan, 430030 China; 2 Department of Surgery, Tongji Hospital, Tongji Medical college, Huazhong University of Science and Technology, Wuhan, 430030 China; 3 Research Center for Bioengineering and Sensing Technology, University of Science & Technology Beijing, Beijing, 100083 China; 4 School of Biomedical Engineering, Health Science Centre, Shenzhen University, Shenzhen, 518060 China

**Keywords:** Nanoparticles, Tumor immunotherapy, Tumor vaccines, Immunogenic cell death, Tumor immune microenvironment

## Abstract

Host-tumor immune interactions play critical roles in the natural history of tumors, including oncogenesis, progress and metastasis. On the one hand, neoantigens have the potential to drive a tumor-specific immune response. In tumors, immunogenic cell death (ICD) triggered by various inducers can initiate a strong host anti-immune response. On the other hand, the tolerogenic tumor immune microenvironment suppresses host immune responses that eradicate tumor cells and impair the effect of tumor therapy. Therefore, a deeper understanding and more effective manipulation of the intricate host-tumor immune interaction involving the host, tumor cells and the corresponding tumor immune microenvironment are required. Despite the encouraging breakthroughs resulting from tumor immunotherapy, no single strategy has elicited sufficient or sustained antitumor immune responses in most patients with specific malignancies due to limited activation of specific antitumor immune responses and inadequate remodeling of the tolerogenic tumor immune microenvironment. However, nanotechnology provides a unique paradigm to simultaneously tackle all these challenges, including effective “targeted” delivery of tumor antigens, sustained ICD mediation, and “cold” tumor microenvironment remodeling. In this review, we focus on several key concepts in host-tumor immune interactions and discuss the corresponding therapeutic strategy based on the application of nanoparticles.

## Introduction

The role of the immune system in tumor development has been increasingly acknowledged. On the one hand, chronic inflammation is linked with tumor initiation, progression and dissemination [1–3]. For example, sufficient evidence has demonstrated the links between viral infection and tumor development, including human papillary virus-related cervical cancer, hepatitis-related liver cancer and Epstein-Barr-related nasopharyngeal cancer [4]. On the other hand, the tumor mass attracts diverse infiltrating immune cells and forms a unique tumor immune microenvironment, which also significantly affects the immunotherapeutic response [5, 6]. However, the detailed mechanism of host-tumor immune interactions and their influences on tumor proliferation, survival and metastasis are only now being discovered.

Tumor immunotherapy was first recognized inadvertently in the late nineteenth century when Coley injected bacterial products into an inoperable sarcoma and observed tumor suppression and concomitant enhanced immunity [7]. Modern immunotherapy strategies have been tremendously advanced owing to fast-paced multidisciplinary progress, including developments in oncology, immunology, molecular biology, pharmacology. However, no single strategy has been perfected to date. For example, although chimeric antigen receptor T-cell immunotherapy (CAR-T) has led to a relatively high remission rate in patients with acute lymphoblastic leukemia (ALL), it still leads to a poor response rate in patients with solid tumors and may induce a “cytokine storm” during treatment [8, 9]. Immune checkpoint inhibitors have been approved by the FDA for treating various advanced tumors, including small cell lung cancer, melanoma and metastatic bladder cancer; However, they may activate the host immune response inappropriately with immune-related adverse events, and they have a relatively poor therapeutic effects on solid tumors [10]. DC-based tumor vaccines have been proven to induce sound immunogenicity in clinical trials, but they have not succeeded in inducing a significant objective clinical response [11].

The investigation of innovative immunotherapy strategies or reasonable combinations of different immunotherapeutic approaches may lead to promising solutions. Nanoparticle (NP)-based tumor immunotherapy has shown tremendous potential. NPs can deliver various molecular cargos, such as tumor antigens and stimulatory immune molecules, to professional antigen-processing cells (APCs), which have led to NP-based tumor antigen vaccination approaches [12, 13]. Moreover, NPs supply new ways to enhance the immune response induced by immunogenic cell death (ICD)-inducing modalities, such as radio-, photo-, and chemotherapy [14, 15]. Similarly, NPs also provide attractive delivery modes to remodel tumor-driven immunosuppressive microenvironments [16, 17]. Here, we focus on the latest research on the mechanism of host-tumor immune interactions and related NP-based immunotherapy.

### Neoantigens and nanovaccine delivery systems

Host natural immune defense prevents and eliminates tumor growth, metastasis and recurrence. Since altered cancer cells are derived from normal cells, antigens presented by cancer cells are mainly self-antigens to which cognate T-cell responses have been suppressed or eliminated through immune tolerance. However, driver mutations can generate cancer-specific neoepitopes that are recognized by an individual’s immune system as foreign antigens, which constitute ideal targets for use in designing cancer vaccines [18]. Sequence-altered proteins, also termed “neoantigens”, can invoke potent host antitumor immune responses compared with randomly mutated antigens, tumor lysates or whole-cell tumor preparations [19, 20].

**Fig. 1:**
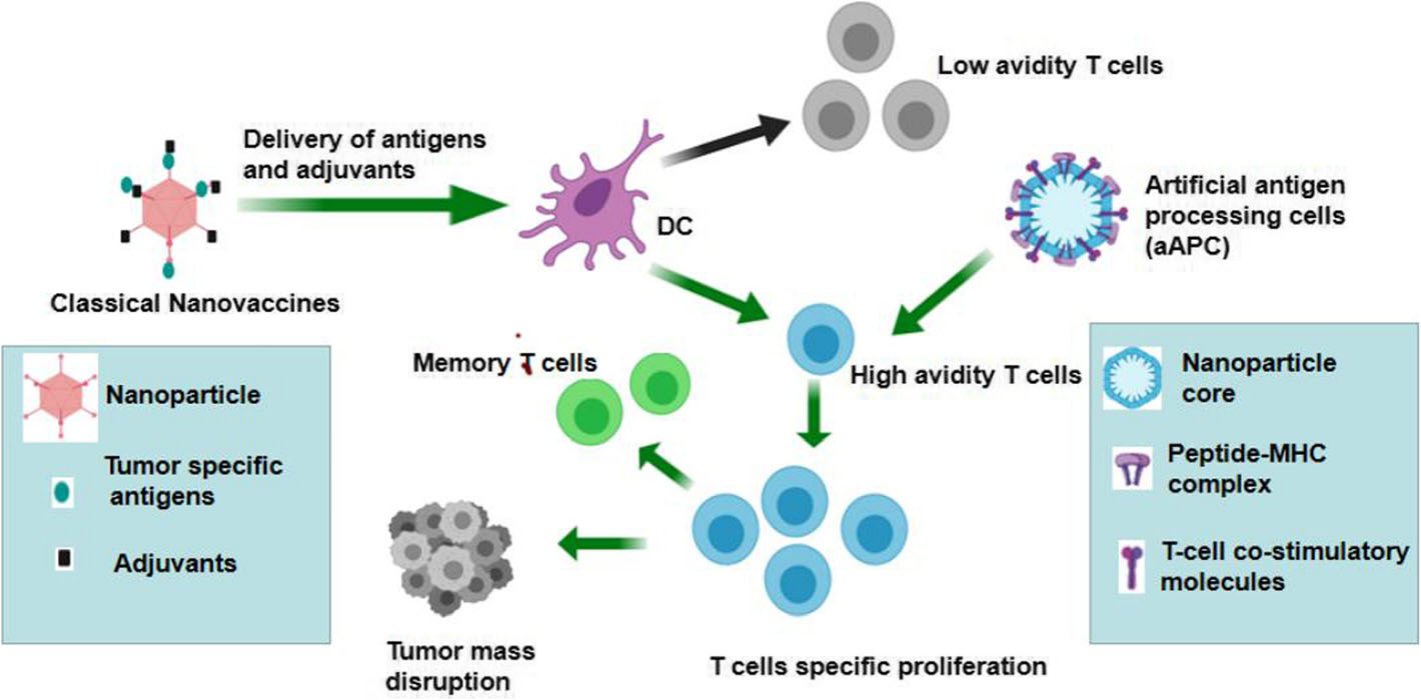
Approach of nanovaccine for successful anti-tumor immune responses. Classical nanovaccines deliver the tumor specific antigens and adjuvants to prime and activate DC cells. Then DC cells induce the proliferation of high avidity T cells, which are the effector cells of anti-tumor immune response. Some specific T cells transform into memory T cells targeting tumor specific antigens. Another type of nanovaccines plays a role as artificial antigen processing cells (aAPC), which can directly induce the specific T cell proliferation through the equipment of peptide-MHC complex and T cell co-stimulatory molecules

In addition to the method of preparing targeting antigens, the delivery and presentation method of tumor-associated antigens (TAAs) to the tumor immune microenvironment (TIM) are also critical for developing efficacious active immunotherapy and improved clinical outcomes [21]. Soluble TAAs are subject to rapid clearance and are poorly taken up by antigen-processing cells (APCs). However, conjugating TAAs onto NPs has been demonstrated to facilitate antigen uptake, cross-presentation and APC maturation [22]. A plethora of therapeutic components, adjuvants and immunomodulatory compounds can be effectively delivered by NP-based platforms, including protein antigens, DNA, and RNA [23]. The approach of nanovaccine stimulation of the antitumor immune response is shown in Fig. 1.

#### Identification of neoantigens and host-tumor immunoediting

Tumor neoantigens, also termed tumor-specific antigens (TSAs), are derived from random somatic mutations in tumor tissue and are not present in normal cells. Based on analyses of neoantigen-specific T-cell reactivity in cancer patients, only a tiny fraction of nonsynonymous mutations in tumors leads to the formation of neoantigens [24, 25]. Although these mutated nonself antigens are recognized by the host immune system and can initiate neoantigen-specific cytotoxic T-cell responses, these antigens are typically less likely to induce specific antitumor immune responses in patients because of insufficient presentation and/or low neoantigen clonal frequency [26, 27]. Importantly, specific T-cell reactions targeting neoantigens are not subject to central or peripheral tolerance and are less likely to induce autoimmunity [19]. Therefore, cancer vaccines against neoantigens are being pursued to elicit robust and specific T-cell responses against cancer.

Currently, continuing efforts are focused on the identification and selection of neoantigens for individual patients [28, 29]. Theoretically, neoantigens can be derived from different mutants of various types, including those with genomic-level, transcriptomic-level, and/or proteomic-level mutations. Several computational methods have been used to identify neoantigens, including high-throughput omics sequencing and high-resolution mass spectrometry (MS) [30]. The affinity of neoantigens to major histocompatibility complexes (MHCs) and the immunogenicity of neoantigens can be further predicted by whole-exome sequencing technology [31]. Undoubtedly, precise individual neoantigen identification is the most important first step for the development of personalized cancer vaccines [19, 32].

The nature of the cancer neoantigen repertoire is substantially influenced by the immune interactions of the developing tumor and host immune system. This interaction has been well documented in preclinical cancer models and has been named “cancer immunoediting” [33, 34]. On the one hand, tumor neoantigens have the potential to induce an autologous T-cell response. Neoantigen-specific CD8^+^ T cells in tumor lesions with a high mutational burden have been observed in clinical patients [35]. On the other hand, the adaptive immune system, especially T cells, has the potential to shape the immunogenicity of developing cancers. Host T-cell immunity in a tumor were shown to silence the expression of certain neoantigens through epigenetic mechanisms in a preclinical model [36]. Increasing the strength of the neoantigen-specific T-cell response is an important way to control tumors. Vaccination with defined neoantigens has been shown to be an effective approach in mouse models [37].

#### NP-delivered neoantigen vaccines to tumors

To elicit robust and potent T-cell responses against cancer, nanovaccines have been extensively investigated for use in neoantigen delivery, as they can protect antigens and adjuvants from degradation, enhance lymphoid organ targeting, and modulate APC functions [13, 38]. Classical multifunctional NPs can stably deliver both tumor antigens and adjuvants to APCs. Tumor-specific antigens engulfed by APCs are also presented via MHC-I to activate cytotoxic CD8^+^ T lymphocytes (CTLs), which are the main effector cells against tumor cells. Moreover, the surface of NPs can also be engineered to include with antigens and costimulatory ligands, which serve as artificial APCs (aAPCs) to initiate antitumoral T-cell immune responses [39, 40]. The use of NPs for antigen delivery and antitumor immune response activation is presented in Fig. 1.

Various NP platforms have been explored for tumor antigen vaccine delivery, including polymeric NPs, liposomes, inorganic NPs, and viral NPs. However, neoantigen-based cancer vaccine delivery with NPs is an emerging area. Nanosized biomaterial delivery systems have been applied to improve the efficacy of peptide- or mRNA-based tumor neoantigen vaccines. For example, a responsive nanovaccine loaded with self-assembling peptide neoantigens and ultra-pH-sensitive polymers can efficiently deliver antigens to the cytoplasm in response to the acidic pH in endosomes, leading to enhanced cross-presentation and a robust antitumor immune response [41]. Liposomes can deliver in vitro transcribed (IVT) mRNA neoantigen vaccines into systemic DCs and induce unusually high levels of antigen-specific CTL responses [42]. It has also been demonstrated that these NP-assisted neoantigen vaccines lead to a further reduction in established tumors when combined with checkpoint blockade antibody treatment [43].

### Immunogenic cell death (ICD) and NP-mediated ICD

Before a lesion progresses into the uncontrolled proliferation of a tumor, the host immune system activate a proactive anticancer immunosurveillance mechanism to reject the neoplastic lesion [44]. However, in very aggressive malignant tumors, cancer cells with a high mutational burden are capable of evading host defense systems by various mechanisms, including acquisition of low immunogenicity, induction of immunotolerance, establishment of immunosuppression, and resistance to immune cell-mediated lysis [45, 46]. During the host-tumor immune interaction, the general immunogenicity of cancerous cells determines the strength of the host anticancer immune response to some degree. Thus, the concept of “immunogenic cell death” (ICD) has emerged in the past few years [47].

The process of ICD induction is stressor dependent. Damage-associated molecular patterns (DAMPs) are released under endoplasmic reticulum (ER) stress or reactive oxygen species (ROS) production, which then act as danger signals to produce immunostimulatory effects [48, 49]. Increasing numbers of novel ICD inducers have been discovered. Compared to conventional approaches, such as chemotherapy or physical therapy, nanotechnology offers an opportunity to enhance their potency to induce ICD while reducing their side effects, although the detailed mechanism is still not clear [14, 50].

#### Definition and features of ICD

**Fig. 2:**
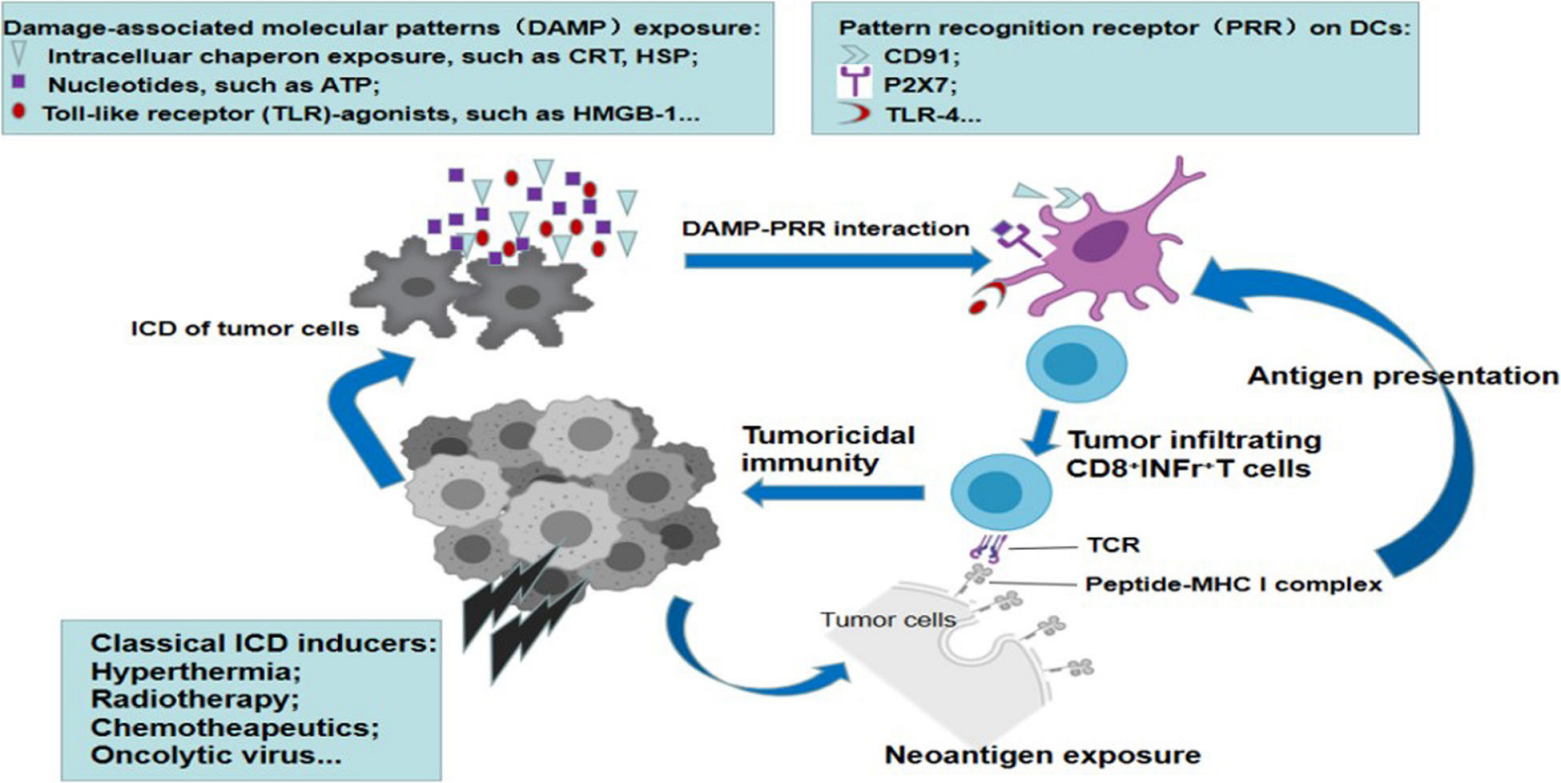
Molecular determinants induced by immunogenic cell death (ICD) of tumor cells and corresponding cell immune response. The tumor releases DAMPs under the stress from classical ICD inducers, such as hyperthermia, radiotherapy, chemotherapeutics, and oncolytic virus. Then the DAMPs interact with PRR on DCs to activate DCs. Tumor cells undergone ICD also expose and present much more neoantigens to DCs. Activated DCs prime the specific T cell response targeting tumor cells to induce antitumoral immunity. The classical DAMP-PRR interaction includes HSP/CD91, ATP/P2X7, HMGB-1/TLR-4

With respect to molecular determinants, three categories of damage-associated molecular patterns (DAMPs) have been found to be associated with the elevated the immunogenicity of dying, stressed or dead cancer cells, i.e., endogenous Toll-like receptor (TLR) agonist, surface exposed chaperones, nucleotides/nucleic acids [51, 52]. Functionally, these DAMPs tend to bind various immune receptors on dendritic cells (DCs) to mediate the maturation of the DCs, which is a crucial step in the stimulation of antitumor immunity. For example, calreticulin (CRT) functions as an “eat me” signal and activates the production of both IL6 and tumor necrosis factor (TNF) by DCs [53]. Heat shock protein (HSPs) can bind CD91 expressed on DCs to assist DC maturation [54]. Secreted ATP can act as a “find me” signal and induce DC maturation and NLRP3 inflammasome activation via purinergic receptors, especially P2X7 [55]. Released high-mobility group box 1 (HMGB1) binds Toll-like receptor (TLR)-4 on DCs to activate the production of proinflammatory cytokines and assist in proper antigen presentation [56]. In addition DAMP interactions with pattern recognition receptors (PRRs) on DCs, neoantigen exposure associated with ICD also activates DCs to induce a tumor-specific T-cell response. The number of tumor-infiltrating effector T cells, such as CD8^+^INFr^+^ T cells is increased, and they recognize and kill tumor cells with immunogenicity via the peptide-MHC I complex. The host immune response induced by ICD in tumors is shown in Fig. 2.

With respect to operational determinants, the following two criteria must be considered [47]. First, the administration of cancer cells succumbing to ICD in vitro into immunocompetent mice can elicit an immune response that protects mice from challenge with live tumor cells of the same type without the use of an adjuvant. Second, ICD in vivo induces signaling that recruits various immune effector cells into the tumor bed, which results in the inhibition of tumor growth via a mechanism that depends, at least in part, on the host antitumor immune response.

ICD is the result of interplay of stressors and cells that requires the elicitation of immunogenic danger signaling and leads to cell apoptosis. Various medicines and therapies can induce ICD, such as specific chemotherapeutics, certain components of Chinese traditional medicine, certain targeted therapeutics, and hypericin-based photodynamic therapy (Hyp-PDT) [57]. ICD inducers can be classified as Type I and Type II according to different pathways of endoplasmic reticulum (ER) targeting. Most of the known ICD inducers are type I ICD inducers, which cause cancer cell apoptosis via non-ER-associated targets but danger signaling via ER targets. This functional split compromises the ability of type I ICD inducers to fully target the ER. In contrast, type II ICD inducers trigger both cell death and danger signaling in a fully ER-directed manner, causing ICD-associated immunogenicity. For example, Hyp-PDT was the first discovered Type II ICD inducer, and it was shown to specifically target the ER in a ‘focused’ manner, thereby inducing a molecularly superior form of ICD [58].

#### NP-mediated ICD and tumor immunotherapy

A subset of chemotherapeutics, including oxaliplatin (OXP), doxorubicin (DOX), and paclitaxel (PTX), has been recognized as a group of classical ICD inducers. Furthermore, nanoparticle (NP)-based chemotherapy has been reported to enhance the antitumor immune response of free ICD inducers via an unclear mechanism. For example, compared with free oxaliplatin (OXP), OXP encapsulated in PLGA-mPEG NPs triggered the release of more DAMPs and induced more dendritic cell and T-lymphocyte infiltration and activation, thereby inhibiting tumor growth in immunocompetent mice [59]. Compared with free doxorubicin (DOX), DOX loaded into highly integrated mesoporous silica NPs further enhanced immunity and antitumor efficacy [60]. Paclitaxel (PTX) and a TLR-4 agonist (SP-LPS) were self-assembled into PLGA NPs that caused enhanced immune cell activation in the tumor microenvironment (TME) and antitumor activity in vivo compared with a Taxol®-treated group [61].

Hyperthermia (HT), such as that induced by microwave thermal ablation, leads to the upregulation of HSPs on the cell membrane and TAAs within tumor cells and is regarded as a classic ICD inducer [62]. Induction of hyperthermia by NPs enables the advantageous control of the thermal dose and localization of the treatment to prevent damaging normal tissues. The typical NPs for HT induction include magnetic NPs exposed to an alternating magnetic field and inorganic light-absorbing NPs induced with near infrared (NIR) light. For example, Fe_3_O_4_ BNF-Starch® magnetic NPs delivered to tumors that were heated to 43 °C for 30 min and exposed to an external magnetic field, triggered an antitumor immune response targeting both the heat-treated primary tumors and unheated distant tumors [63]. Intratumoral injection of Au-DNA hydrogel nanoparticles followed by laser irradiation induced HSP70 upregulation in the tumor and TAA-specific IFN-γ production in splenocytes, significantly inhibiting tumor growth and extending the survival time of tumor-bearing mice [64].

Specific forms of radiation therapy also induce ICD in situ with surface exposure to CRT and release of Heat Shock Protein70 (HSP70) and High Mobility Group Box-1 (HMGB-1), thereby stimulating DC maturation and inducing IFN-γ-producing T cells in vitro and in vivo [65]*.* Nanotechnology allows nanovectorized ionizing radiation to enhance the antitumor immune response in a designable fashion. For example, lipid nanocapsules loaded with a rhenium-188 lipophilic complex used during the fractionated internal radiation of glioblastoma and hepatocellular carcinoma models led to increased cytokine (IL-2 and IFN-γ) production, increased inflammatory cell infiltration, and increased expression of MHC class I and class II in tumor sites [66, 67].

### The tumor immune microenvironment (TIM) and NP-based remodeling strategies

Tumor microenvironments are elaborate conglomerations of transformed cells, blood vessels, fibroblasts, and immune cells. With an increasing understanding of the immunosuppressive nature of the tumor microenvironment during tumor-host interactions, various immunotherapies for cancer are being discovered [68]. Similarly, a positive response to tumor immunotherapy is also dependent on immunomodulatory interactions between tumor cells and the tumor microenvironment [69]. Therefore, in addition to traditional treatment targeting cancer cells directly, remodulating the tumor immune microenvironment (TIM) is crucial for the effective treatment of specific tumors.

Nanomaterials provide attractive delivery modes to target the tumor immune microenvironment. By interacting with various components or pathways in the TIM, NP-based strategies can turn “cold” tumors into “hot” tumors [16, 70]. NPs not only can enhance the host antitumor immune response but can also improve the immune response to immunotherapies.

#### Classifications and characteristics of the TIM

Based on the composition of the immune infiltrate, cellular status, spatial information and the character of the inflammatory response, various subclasses of TIMs have been described [71, 72]. However, ongoing higher-resolution techniques are required to improve TIM classifications and determine the heterogeneity of TIMs in terms of their immunological composition, spatial distribution and function.

According to the total number and type of cells present in the tumor core, TIMs are categorized as infiltrated-excluded (I-E) TIMs and infiltrated-inflamed (I-I) TIMs [71, 73]. I-E TIMs are populated with immune cells but are nearly void of cytotoxic lymphocytes (CTLs). Poor infiltration of CTLs and inactivate markers are characteristics of immunological ignorance; therefore, tumors classified in I-E TIMs are considered to be poorly immunogenic or “cold”. However, tumors in I-I TIM are considered to be immunologically ‘hot’ tumors and are characterized by high infiltration of lymphocytes and PD-1^+^ CTLs. In the ‘hot’ tumor subclass, tumor cells express higher immune-dampening PD-L1 and have higher responses to immune checkpoint blockade (ICB) therapy. As the spatial information of immune cells in the TIM was further revealed, a tertiary lymphoid structure (TLS) in TIMs was identified. A TLS-TIM contains a variety of lymphocytes, including naive and activated conventional T cells, regulatory T (Treg) cells, B cells and dendritic cells (DCs). In contrast to I-I TIMs, TLS-TIMs are generally present at the invasive tumor margin or in the stroma with lymphoid recruitment and immune activation, and they are typically formed in settings of enhanced inflammation, such as after administration of tumor vaccines or immunotherapy. However, TLS-TIMs are often, but not always, correlated with a positive prognosis.

Considering whole-exome sequencing (WES) and gene expression profiling (GEP) data obtained from fresh surgical specimens taken from cancer patients, Project High-tech Omics-based Patient Evaluation (HOPE) classified TIM immunological status into 4 types according to the expression level of PD-L1 and CD8B genes; i.e., type A TIMs express PD-L1^+^ CD8B^+^; type B TIMs express PD-L1^+^ CD8B^−^; type C TIMs express PD-L1^−^ CD8B^−^; and type D TIMs express PD-L1^−^ CD8B^+^. Type A TIMs, with PD-L1^+^ and CD8B^+^, significantly promote antitumor immune responses and are distinguished by the upregulation of cytotoxic T lymphocyte (CTL)-associated genes, T-cell-activating genes, dendritic cell (DC) maturation genes, and T-cell-attracting chemokine genes. In contrast, type C TIMs, expressing PD-L1^−^ and CD8B^−^, exhibit downregulated T-cell-activating genes and upregulated cancer driver genes, which suggests an immune-suppressive status. More detailed characteristics of TIM would be very helpful to evaluate the immunological status of the tumor and predict the antitumor response of specific immunothrapy [74, 75].

#### TIM resistance to the host antitumor immune response and NP-mediated remodeling of the TIM

**Fig. 3:**
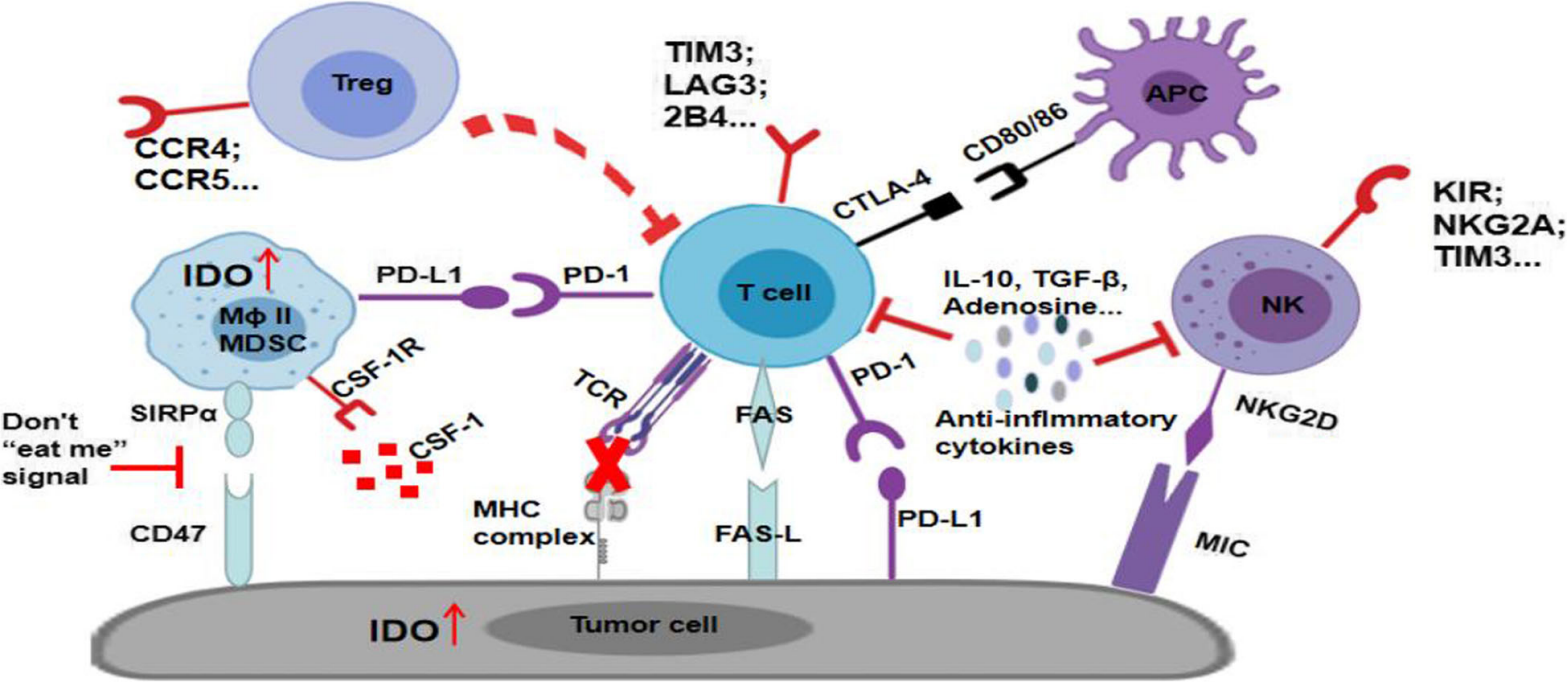
Immunosuppressive milieu in the tumor immune microenvironment (TIM). On the tumor side, tumor cells could evade the attack from T effector cells (Teff) by concealing the MHC complex, inhibit the function of Teff by up-regulation of PD-L1, and induce the apoptosis of Teff by up-regulation FasL. Some metabolic pathways related to immune suppression also increased in tumor cells, such as IDO. The up-regulation of CD47 plays as a “Don’t eat me” signal to evade the phagocytosis of macrophages via CD47/SIRPα. For T cells in the TIM, suppressive immune checkpoint molecules were upregulated, including PD-1, CTLA-4, TIM3, LAG3, 2B4. For NK cells in the TIM, upregulated immunosuppressive molecules include KIR, NKG2A, TIM3. For Treg cells, chemokine receptors upregulation contributes to the infiltration of Treg and the role of immunomodulation. Many other immunomodulatory molecules also contributes to the suppressive TIM. For example, IL-10,TGF-β, adenosine could play as anti-inflammatory cytokines to suppress the function of T cells and NK cells in the TIM; Colony-stimulating factor-1 (CSF-1) could recruit more M2 macrophages (Mϕ II) and myeloid-derived suppressor cells (MDSC)

Tumor genotypes and phenotypes dynamically shape the TIM. Eventually, immune evasion is established during the interactions between the host immune system and cancer cells [76–78]. From the perspective of the tumor, the mechanism of immune evasion includes PD-L1 upregulation to induce T-cell exhaustion, Fas-L upregulation to induce T-cell apoptosis, low expression of the MHC complex to decrease TCR stimulation, and upregulation of CD47 to induce the “Don’t eat me” signal of macrophages. From the host perspective, the mechanism of TIM resistance to host immune response or immunotherapy is the subject of ongoing research, and includes the dysfunction of antitumor effector cells (CTLs, M1 macrophages, and NK cells), insufficient antigen presentation by DCs, activation of regulatory or suppressor cells (Treg, M2 macrophages, and/or myeloid-derived suppressor cells) and other inhibitory soluble mediators (cytokines and enzymes). The immunosuppressive milieu during host-tumor interactions in the TIM is depicted in Fig. 3. All of the molecular pathways shown can be potential therapeutic targets for tumor immunotherapy.

T cells constitute the key effector cells of the acquired immune response. The T-cell transition from an effector (Teff) cell to an exhausted (Tex) cell is a major contributing factor in the failure of host immune elimination of tumors and is accompanied by the increased expression of exhaustion-associated molecules, such as LAG3, 2B4 and TIM3 [79, 80]. Negative costimulatory molecules, also termed immune checkpoints, suppress the immune response mediated by TCR signaling, including PD-1/PD-L1, CTLA-4/CD80/CD86, and Tim-3/Galectin-9 signaling. Various immune checkpoint inhibitor (ICI) therapies have already been applied in the clinic. However, in addition to a relatively low clinical response rate, immune checkpoint inhibitor (ICI) therapy inevitably leads to adverse effects during systemic administration [10]. However, nanotechnology can address these issues. An engineered plasmid DNA coding a protein antagonist of PD-L1 called a “trap” was designed and loaded in lipid protamine-DNA (LPD) NPs. These NPs enabled local in situ expression of the PD-L1 antagonist, lowering the systemic toxicity induced upon administration [81].

Dendritic cells (DCs) are regarded as professional antigen-processing cells (APCs) and play crucial roles in initiating and activating the tumor immune response. DCs consist of immature dendritic cells (imDCs) and mature dendritic cells (mDCs). mDCs are likely to induce Th1-type immune responses through the activation of tumor-specific cytotoxic T lymphocytes (CTLs) [82]. However, under immunosuppressive conditions, such as those in the TIM, the ability to mature and present antigens is inhibited, resulting in a tolerogenic DC type [83, 84]. NPs have different potentials to modulate reactive oxygen species (ROS) and affect the functional status of DCs. For example, TiO2 NPs have oxidative characteristics to promote DC maturation and a Th1-based response [85].

Tumor-associated macrophages (TAMs) are important immunoregulatory cells that infiltrate the tumor stroma, performing the same kind of function as alternatively activated M2 macrophages. TAMs with M2 phenotypes are capable of secreting immunosuppressive cytokines and growth factors that inhibit CTL infiltration in tumors, promoting tumor cell growth and angiogenesis and facilitating tumor invasion and metastasis [86, 87]. NPs have several ways to target TAMs. Lipid NPs with a siRNA payload have been developed to modulate the expression of chemokine receptor CCR2 in inflammatory monocytes, thereby selectively inhibiting the recruitment of these cells and TAMs into the TIM [88]; Iron oxide NPs possess the intrinsic ability to polarize immunosuppressive TAMs with an M2 phenotype towards the activated M1 phenotype, which can activate the inflammatory response and inhibit tumor growth in the presence of Th1 cytokines [89].

Myeloid-derived suppressor cells (MDSCs) represent a group of undifferentiated bone marrow-derived heterogeneous cell populations that coexpress Gr-1 and CD11b [90, 91]. Similar to macrophages, MDSCs can be polarized into the M1 or M2 phenotype, characterized by cytokine secretion of Th1 (e.g., IL-12, IFN-γ, and TNF-α) or Th2 (e.g., IL-10 and TGF-β) cells. Two cationic polymers, cationic dextran and polyethyleneimine, have shown the ability to repolarize MDSCs from the M2 to M1 phenotype by modulating Toll-like receptor (TLR) signaling, thereby improving the efficacy of cancer immunotherapy [92]. Another strategy involves using NPs to inhibit the function and development of MDSCs by means of specific binding to scavenger receptor type B 1 (SCARB1), a receptor with high affinity for high-density lipoprotein (HDL). Synthetic HDL biomimetic NPs were prepared for MDSC inhibition and substantially retarded tumor growth in a murine B16F10 melanoma model [93].

Various cytokines in the TIM can dictate the extent and phenotype of immune factor infiltration, including interleukins (ILs), interferons (IFNs), tumor necrosis factors (TNFs), colony stimulating factors (CSFs), and various chemokines [94]. Disequilibrium between proinflammatory and anti-inflammatory cytokines in the TIM is a direct reflection of host-tumor immune interactions [95]. Traditional cytokine therapy shows unsatisfactory effects, which have been ascribed to the presence of numerous immunosuppressive cytokines and poor tumor site targeting. NPs are likely to provide a productive strategy for blocking the immunosuppressive effects of tumor-promoting cytokines and/or enhancing the immunostimulatory effects of antitumor cytokines, thus increasing the antitumor immune response. For example, the combined delivery of a TGF-β antagonist and IL-2 by nanoscale liposomal polymeric gels substantially enhanced activated CD8^+^ T-cell infiltration in tumors and improved the survival of metastatic melanoma-bearing mice [96]. Downregulation of TGF-β with liposome-protamine-hyaluronic acid (LPH) NPs loaded with TGF-β siRNA led to an increase in the tumor-infiltrating CD8^+^ T-cell ratio and a decrease in regulatory T-cell levels, thereby inhibiting tumor growth significantly [97].

Some metabolic enzymes have been demonstrated to participate in immune modulation. For example, indoleamine-2,3-dioxygenase (IDO) catalyzes tryptophan into canine urea, which participates in immune tolerance through the inhibition of T cells and NK cells and the activation of Tregs and MDSCs [98]. Considering the primary role of IDO in tumor immune tolerance, IDO the use of inhibitors have been proposed as an immunotherapy strategy. However, monotherapy with IDO inhibitors in cancer immunotherapy has been limited [99]. NP-mediated synergistic immunotherapeutic strategies have shown good performance. For example, a self-assembled nanocapsule loaded with the IDO inhibitor 1-MT modified with hyaluronic and an anti-PD-1 antibody achieved potent antitumor efficacy in a B16F10 mouse melanoma model [100]. Mesoporous silica NPs used for delivering a lipid-conjugated IDO inhibitor prodrug plus oxaliplatin caused tumor regression or eradication in a pancreatic ductal adenocarcinoma mouse model, wherein the IDO inhibitor prodrug showed the unexpected benefit of synergistically enhancing the ICD response upon treatment with oxaliplatin [101].

### Optimization strategy for using NP-based tumor immunotherapy

Although advances in nanotechnology, and thus in NP-based immunotherapy, has led to many breakthroughs, several challenges to their use in clinical treatments remain. Delivery of therapeutic components to target cells is a determinant step for therapeutic efficacy. How to improve the inefficient tumor penetration and non-selective targeting was the prospect for optimal NPs based immunotherapy [102]. At the same time, design of multi-functional NPs or synergistic therapeutic regimen was also a promising way for improved clinical benefit [103]. Optimizing approaches for NPs-based tumor immunotherapy are shown in Table 1.

**Table 1:** Optimizing approaches for NPs-based tumor immunotherapy

NPs performance for optimizing	Common tuning factors	Mechanism	Examples of optimizing strategy	References
NPs penetrability	Size of NPs	Switch PRs to small particles once accumulating at the tumor site for deep penetration	Size shrinkage triggered by acid PH, overexpressed enzyme, redox condition,or reactive oxygen species (ROS)	[104–106]
	Dense tumor matrix	Functionalizing NPs with enzymes that degrade tumor ECM components	Conjugate bromelain to mesoporous silica nanocarrier; Coupling hyaluronidase to the surface of PLGA-NPs	[107, 108]
NPs selectivity	Tumor selectivity	Switch PRs to larger particles once accumulating at the tumor site for longer retention	Initial nanoparticles with relatively small sizes form aggregations which induced by internal stimuli (such as enzymes, PH and redox), or triggered by external stimuli (such as light, temperature)	[105]
		Coating with NPs with biologically derived cell membranes	Erythrocyte membrane-enveloped PLGA-NPs;Cancer cell membrane coated PLGA-NPs	[109, 110]
		Coupling tumor homing peptide on the surface of NPs	Coupling iRGD on the surface of NPs	[111]
	Cellular selectivity	Attaching a ligand or antibody fragments that are specific for the targeting cell	CD40-coupled PLGA-NPs targeting DC cells; G5-methotrexate nanoparticles targeting the folate receptor-2 (FOLR2) on tumor associated macrophage	[112, 113]
	Intracellular selectivity	Tuning the surface chemistry of NPs to facilitate the escape of endolysosomal pathway	Cationic particle surfaces facilitate NPs to release into the cytosol, rely on the “proton sponge effect” at low PH	[114]
		Incorporate cell-penetrating peptides on the NPs to enhance the intracellular delivery	Incorporate cell-penetrating peptides KALA on the NPs surface	[115]
NPs versatility	Sensitive NPs to external stimuli	Take advantage of features of sensitive NPs to develop combination therapy	Radio-sensitive NPs for radiotherapy; photo-sensitive NPs for phototherapy; magnetic-sensitive NPs for magnetodynamic therapy	[116–118]
	Theranostic NPs	Combine the function of imaging and therapy for some NPs	Theranostic nanoreactor for imaging-guided combined tumor therapy;Theranostic applications of nanodiagnosis and nanotherapy with some NPs, including polymeric NPs, quantum dots, magnetic NPs	[119–121]
	Synergistic immunotherapy	Develop the multi-functional NPs or combine the present therapy methods with NPs based immunotherapy	Combine other classical immunotherapy and molecular targeting treatment, such as PD-1/PD-L1 antibody treatment	[16, 122]

#### Optimizing NP penetrability in tumor immunotherapy

NPs can preferentially accumulate at a tumor site through the enhanced permeability and retention (EPR) effect. However, inefficient NP delivery is a critical obstacle to overcome to realize optimal therapeutic efficacy because of the contradiction between tumor penetration and tumor retention [123]. As NPs rely on slow diffusion for intratumoral transport, physicochemical properties such as size, shape, and surface charge have profound effects on NP delivery. The ideal size of a NP greatly defines the equilibrium point between tumor penetration and tumor retention [104]. Theoretically, the smaller the NPs are, the faster they are transported within the tumor. However, very small particles (< 5.5 nm) are eliminated more quickly by renal tissue, which compromises their tumor retention. Therefore, the choice of NP size requires a balance between the circulation time in blood vessels and the penetration depth in the tumor parenchyma. To optimize this situation, various size-tunable NPs have been designed with improved delivery efficacy [105]. Internal stimuli (enzymes, pH, and redox) and external stimuli (light and temperature) have been introduced to design a series of stimulus-induced aggregation and shrinkage strategies for size-switching NPs [104, 106]. For tumor nanovaccines, lymph nodes are the sites of antigen-presenting cell (APC) priming and subsequent adaptive immune response activation. In general, NPs larger than 50–100 nm tend to become trapped in the extracellular matrix and cannot freely drain in the lymphatic system but can be phagocytosed and transported to lymph nodes by DCs or monocytes in the tissue. However, NPs smaller than 6 nm tend to drain into the blood, not the lymphatic system [124, 125]. Therefore, the appropriate size of nanovaccines must be considered for optimal lymph node targeting.

Apart from physicochemical tuning of NPs for optimal delivery, peptide and chemical modifications to the NPs surface have been developed to improve the tissue penetration [126, 127]. For example, iRGD, a tumor-penetrating peptide which binds to αv integrins expressed on tumor endothelial cells and other cells in tumors, but not in normal tissues,enhanced intratumoral entry of attached co-applied NPs [111]. And CSG, a newly discovered peptide which binds to laminin–nidogen complexes in the extracellular matrix (ECM) of tumor, with little or no peptide detected in normal tissues, can improve the perfusion and intratumoral uptake of iron oxide NPs [128]; A dense network of highly disorganised ECM often encountered in aggressive and treatment-resistant cancers. ECM-degrading strategy has also been realized by functionalizing NPs with enzymes that degrade tumor ECM components [107, 108]. For example, Coupling the recombinant human hyaluronidase PH20 (rHuPH20) on the surface of poly (lactic-co-glycolic acid)-b-polyethylene glycol (PLGA-PEG) NPs can facilitate efficient tumor penetration [108]. However, it should be noted that the tumor ECM degradation may cause detrimental effect on tumor metastasis via facilitating the leaking out of the tumor cells.

#### Optimizing NP targeting ability in tumor immunotherapy

A major hurdle to achieving therapeutic treatment by nanoimmunotherapy is the appropriate release of active therapeutic agents at the desired site, which involves protecting NPs from degradation and clearance while in the blood circulatory system, facilitate NP homing to particular target cells, and NP transport to the proper intracellular compartment.

Nonspecific uptake by the reticuloendothelial system (RES) in the circulation significantly affects NP therapeutic efficacy [129]. The tuning of physicochemical features has been demonstrated to decrease the incidence of off-target side effects. For example, shape has a significant effect on the biodistribution, clearance and biocompatibility of NPs in vivo. Long-rod mesoporous silica NPs (MSNs) have a slow rate of excretion compared to short-rod MSNs [130]. To evade RES elimination and immunological surveillance, NPs camouflaged by biologically derived cell membranes have recently attracted attention. During systemic circulation, vesicle membrane from different cell types (such as red blood cells, platelets, macrophages, and cancer cells) are expected to act as a concealing cloak against opsonization and RES clearance and as a tumor-homing navigator to enhance tumor accumulation [109, 110]. For example, compared to PEGylated NPs with half-lives (half of the time before elimination) of 15.8 h, red blood cell membrane-coated NPs exhibit a half-life of 39.6 h, a twofold improvement [131].

For cellular selectivity of NPs, One common strategy is attaching a ligand or peptide that facilitates homing to a particular target cell to enhance NPs immunotherapy [112, 132]. Take DC cell targeting as an example, specific monoclonal antibodies targeting DCs cell-surface molecules were coupled to NPs for the specific delivery of antigens and adjuvants to DCs. For example, CD40-coupled PLGA-(Ag/TLR3 + 7 L) NPs was more efficiently targeted to and internalized by DC, and stimulating much more potent CD8^+^ T cell responses after subcutaneous vaccination, compared to the non-targeted NPs [113]. Antibody fragments specific for cell surface antigens have also been used for the targeting cell delivery. For example, F (ab′)_2_ fragments against unique cell surface antigens expressed only by the adoptively transferred T-cells have been used for conjugating the PEGylatedliposomes with IL-2, which improve the efficacy of targeting NPs to adoptively transferred T-cells in healthy animals and in a model of metastatic melanoma [133].

For intracellular selectivity, NPs escape the endolysosomal pathway into the cytoplasm is required during NPs-based therapy [134]. The size, shape and surface chemistry of NPs affect the endocytosis pathways. For example, cationic particle surfaces facilitate NPs disrupt the intracellular vesicle compartment and release into the cytosol, rely on“proton sponge effect” at low PH [114]; Another way of enhancing the intracellular delivery is to incorporate cell-penetrating peptides on the NPs surface. For example, conjugation of KALA, one PH sensitive cell-penetrating peptide, increased the immunostimulatory abilities of lipid NPs when compared to soluble CpG or NPs without KALA [115].

#### Optimizing NP versatility in tumor immunotherapy

The incorporation of multiple functions into an NP system is highly beneficial for clinical translation. On the one hand, specific NPs may have unique features that facilitate the treatment of tumors, such as radiosensitive NPs for radiotherapy, photosensitive NPs for phototherapy, and magnetic-sensitive NPs for magnetodynamic therapy [116–118]. In addition, some innovative NPs, such as magnetic NPs, gold NPs, and quantum dots, which enable both imaging and therapeutic functions simultaneously, are emerging as theranostic NPs [119–121]. On the other hand, tumors have microenvironments that differ from those of normal tissue, and we can take advantage of these features to design NPs with improved tumor targeting, such as pH-sensitive NPs, enzyme-sensitive NPs and reduction-sensitive NPs [135, 136]. Moreover, many other therapeutic components have been developed that synergize with NP-based tumor therapy, such as neovascularization blockers, oxygen-producing compounds and nitric oxide (NO) generation compounds [16, 122].

## Conclusions

With in-depth research on tumor biology, tumor immunology, and molecular biology, the mechanism of host-tumor immune interactions has been increasingly elucidated. Against this background, advances in nanomedicine further drive the development of NP-based tumor immunotherapy. In this review, several key points and NP-based solution strategies are discussed. First, neoantigens are linked to the immunogenicity of tumors. Nanovaccines targeting neoantigens can elicit a stronger specific immune response and more stable therapeutic effects than traditional tumor vaccines. Second, immunogenic cell death is recognized as the critical driving force for initiating the antitumor immune response. NP-mediated ICD compounds provide a new modality to enhance immunotherapy. Third, the tumor constitutes a tolerogenic immune microenvironment. NP-based immunotherapy has enormous potential for remodeling the TIM. Up to date, in the field of nanoimmunotherapy, several NP-based compounds have been used in clinical trials. These compounds strengthen the anti-tumor immunity in one or more of mechanisms as described in Table 2.

**Table 2:** Ongoing clinical trials of nanoimmunotherapy for cancer

Compound name	NP formulation	NP payloads	Mechanisms of immunotherapy	Anti-tumor immunological effect	Clinical trials	Reference
RNA-LPX	Lipoplexes	RNA encoding mutant neo-antigens	NP-based tumor RNA delivery	Systemic DC targeting; Adaptive T cell response	Phase I	[137]
JVRS-100	Cationic liposome	Plasmid DNA Complex	NP-based tumor DNA delivery	Anti-tumor immune stimulation	Phase I	NCT00860522
Anti-EGFR-IL-dox	Liposome	Doxorubicin-loaded anti-EGFR	NP mediated ICD	Immunological cell death induction	Phase II	NCT02833766
PTX-LDE	Lipid NPs	Paclitaxel	NP mediated ICD	Immunological cell death induction; DC maturation	Phase II	[138]
NBTXR3	Hafnium oxide NPs	Anti-PD1	NP mediated ICD	Immunological cell death induction	Phase I	NCT03589339
Ferumoxytol	Iron oxide NPs	Nil	NP mediated remodeling of TIM	Polarizing M2 to M1	FDA approved for anemia and kidney disease	[89]

In addition to the potential toxicity of nanomaterials, the clinical application of NP-based tumor immunotherapy requires overcoming several challenges, such as the penetrability and selectivity of NPs. However, these limiting factors can be optimized by tuning physiochemical features or by functional modification. Finally, the development of tumor therapies is a multidisciplinary field. NP-based tumor immunotherapy should be part of personalized and synergistic treatment regimens.

## Data Availability

Not applicable as no datasets were generated or analyzed.

## Abbreviations

ACC: Acute lymphoblastic leukemia
APCs: Antigen processing cells
CAR-T: Chimeric antigen receptor T-cell
CTL: Cytotoxic T lymphocytes
DAMPs: Damage-associated molecular patterns
DC: Dendritic cells
ECM: Extracellular matrix
EGFR: Epidermal growth factor receptor
ER: Endoplasmic reticulum
ICB: Immune checkpoint blockade
ICD: Immunogenic cell death
MSN: Mesoporous silica nanoparticles
NO: Nitric oxide
NP: Nanoparticle
ROS: Reactive oxygen species
TAAs: Tumor-associated antigens
TIM: Tumor immune microenvironment
TSA: Tumor-specific antigens
